# Research and Analysis of MEMS Switches in Different Frequency Bands

**DOI:** 10.3390/mi9040185

**Published:** 2018-04-15

**Authors:** Wenchao Tian, Ping Li, LinXiao Yuan

**Affiliations:** School of Electro-Mechanical Engineering, Xidian University, Number 2 Taibai South Road, Xi’an 710071, China; wctian@xidian.edu.cn (W.T.); lxyuan@stu.xidian.edu.cn (L.Y.)

**Keywords:** microelectromechanical systems (MEMS) switch, isolation, insertion loss, dielectric charging, contact failure, temperature-stable

## Abstract

Due to their high isolation, low insertion loss, high linearity, and low power consumption, microelectromechanical systems (MEMS) switches have drawn much attention from researchers in recent years. In this paper, we introduce the research status of MEMS switches in different bands and several reliability issues, such as dielectric charging, contact failure, and temperature instability. In this paper, some of the following methods to improve the performance of MEMS switches in high frequency are summarized: (1) utilizing combinations of several switches in series; (2) covering a float metal layer on the dielectric layer; (3) using dielectric layer materials with high dielectric constants and conductor materials with low resistance; (4) developing MEMS switches using T-match and π-match; (5) designing MEMS switches based on bipolar complementary metal–oxide–semiconductor (BiCMOS) technology and reconfigurable MEMS’ surfaces; (6) employing thermal compensation structures, circularly symmetric structures, thermal buckle-beam actuators, molybdenum membrane, and thin-film packaging; (7) selecting Ultra-NanoCrystalline diamond or aluminum nitride dielectric materials and applying a bipolar driving voltage, stoppers, and a double-dielectric-layer structure; and (8) adopting gold alloying with carbon nanotubes (CNTs), hermetic and reliable packaging, and mN-level contact.

## 1. Introduction

With the development of communication technology, electronic products are gradually becoming more and more miniaturized and multifunctional. Due to a series of advantages, such as miniaturization, intelligence, multifunction, and high integration, microelectromechanical systems (MEMS) are widely used in the military and civil fields [1]. The MEMS switch is one of the most basic components in radio frequency (RF) devices. It is also the core of RF circuits. It can be used alone or in combination with other microwave circuits or devices to form other composite devices, such as phase shifters and filters and reconfigurable antennas [2,3,4,5]. Compared with the traditional positive-intrinsic-negative (PIN) and field-effect transistor (FET), the MEMS switch has excellent performance in terms of insertion loss, isolation, frequency, and linearity and has been widely used in satellite communications, navigation, and radar systems [6,7].

Nowadays, most MEMS switches are researched in the centimeter band (3–30 GHz). However, with the continuous development of communication technology, the requirements for wireless bandwidth have become increasingly prominent, and the original communication frequency band has become very crowded and is unable to meet the exponential growth of communication bandwidth requirements. As a result, the communication system has gradually shifted toward the millimeter wave (30–300 GHz) and submillimeter wave (300–3000 GHz) development, and communications and transmission technologies in these bands have become the current research hotspot. MEMS switches have excellent performance and are widely used in low frequency. However, the parasitic effect of the switch is significantly enhanced as the frequency increases, resulting in a drastic deterioration of the isolation performance. At the same time, the skin effect of the conductor is also significantly increased in high frequency, so the insertion loss performance is dramatically worsened [6]. In addition, MEMS’ reliability issues caused by dielectric charging, contact failures, temperature instability, etc. [8,9] and packaging [10,11,12] are the main reasons limiting their application in high-frequency fields. 

This paper summarizes the research status of MEMS switches in the centimeter, millimeter, and submillimeter bands and some reliability issues, such as dielectric charging, contact failure, and temperature instability. The performance parameters in different bands and reliability issues are analyzed to provide research ideas for implementing high-performance MEMS switches in high-frequency fields.

This remainder of this paper has four parts: Section 1 mainly introduces the application background of MEMS switches and the necessity of MEMS switch research in different frequencies. Section 2 presents brief introduction to MEMS switches. Section 3 presents a brief introduction to MEMS switches and summarizes the research status of MEMS switches in different frequency bands and certain reliability issues, such as dielectric charging, contact failure, and temperature instability. Section 4 draws conclusions based on Section 3 and summarizes some methods to achieve high-performance MEMS switches in high frequency. The last section summarizes the work of the full text.

## 2. Brief Introduction to MEMS Switches

MEMS switches can be classified into capacitive shunt switches [13], capacitive series switches [14], direct current (DC) shunt switches [15], and DC series contact switches [16] based on the method of contact. The DC contact switches (shunt and series) show better isolation and insertion loss at a wide frequency from DC to gigahertz (GHz). However, the isolation is related to the parasitic capacitance, and the insertion loss depends on the contact resistance. In addition, the dielectric materials are much harder than the contact materials, which are typically gold (Au) or other metals, and friction and adhesion happen frequently in the DC contact switches. The capacitive series switches present better isolation and insertion loss in the frequency range of several GHz (typically 1–10 GHz), and the capacitive shunt switches show better isolation and insertion loss at higher frequency ranges (typically tens of GHz). Furthermore, the current MEMS switches can also be classified into piezoelectric [17], electrothermal [18], magnetic [19], and electrostatic [20] driving according to their actuation mechanisms. Magnetic and electrothermal actuations exhibit the good performance of high-contact force and low-actuation voltage, but the switch time is a little longer, and they consume more power [21,22]. Piezoelectric actuation shows fast switch time and low-actuation voltage, but parasitic actuation occurs frequently due to the mismatch in the coefficients of thermal expansion [21]. Electrostatic actuation MEMS switches are most commonly used for their advantages of high isolation, low insertion loss, and low power loss [21]. Shunt capacitive MEMS switches are the most researched because of their excellent electromagnetic performance at high frequency ranges. 

**Figure 1:**
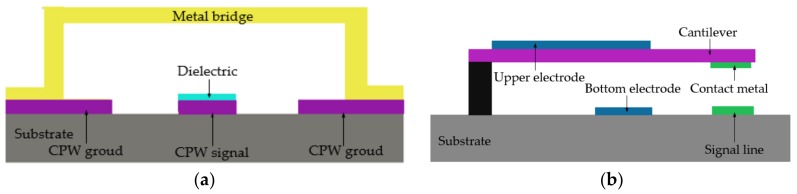
The schematics of typical microelectromechanical systems (MEMS) switches: (**a**) shunt capacitive MEMS switch; (**b**) DC contact switch. Coplanar waveguide (CPW) = coplanar waveguide.

The typical schematic structure of a standard shunt capacitive MEMS switch is shown in Figure 1a [23,24]. The switch contains a silicon substrate, a silicon nitride insulator, a metal bridge, a coplanar waveguide (CPW) signal line, and two CPW ground lines. The movable bridge moves toward the dielectric layer when the pull-down voltage is applied. Then, the RF signal in the CPW signal line is isolated through the metal bridge because of the large capacitance between the CPW signal line and the metal bridge after the contact between the dielectric layer and metal bridge. When the pull-down voltage is removed, the metal bridge pulls up due to its elastic restoring force. Then, the RF signal passes through the signal line because of the much smaller capacitance between the CPW signal line and the metal bridge in this state. Figure 1b shows the schematic structure of the DC contact switch. The switch is in the “ON” state when the cantilever is in the “Down” state, which is the opposite of shunt capacitive MEMS switch.

## 3. The Research Status of MEMS Switches in Different Frequencies

This section will summarize the research status of MEMS switches in different frequency bands (centimeter, millimeter, and submillimeter) and their reliability (dielectric charging, contact failures, and temperature instability). Finally, some multiband switches are introduced. The research on different bands focuses on the insertion loss and isolation of MEMS switches. Due to the wide band of the millimeter wave, it has been divided into the Q band (30–50 GHz), V band (50–75 GHz), and W band (75–110 GHz).

### 3.1. Research Regarding Insertion Loss and Isolation of MEMS Switches in Different Bands

Insertion loss and isolation are two critical performance parameters that are determined by the structures and materials of MEMS switches [6]. Insertion loss means the ratio of the input signal to the output signal when the switch is ON. Isolation means the ratio of the input signal to the output signal when the switch is OFF. There have been numerous studies to achieve high isolation and low insertion loss in different frequency bands. 

#### 3.1.1. MEMS Switches with Serpentine Flexure Structures in Different Frequency Bands

Serpentine flexure structures reduce the spring constant and increase the series inductance of the MEMS bridge, thereby achieving high isolation and low-actuation voltage. Table 1 summarizes the performance parameters of MEMS switches using the bridges with serpentine flexure structures in different frequency bands recently. 

MEMS switches with serpentine flexure structures in centimeter band have been reported in [25,26,27,28]. Tang et al. [25] designed a single-bridge MEMS capacitive switch and a double-bridge MEMS capacitive switch with serpentine folded suspensions to achieve higher isolation at X-band frequencies, and the measurement results presented an isolation of 16.5–28 dB for the single-bridge switch and 25–35 dB for the double-bridge switch at 10–13 GH. Kim et al. [26] designed a MEMS switch with a bridge supported by four springs, and it achieved an insertion loss of 0.19 dB at 10 GHz and 0.2 dB at 15 GHz and an isolation of 27.17 dB at 10 GHz and 23.57 dB at 15 GHz. Czaplewski et al. [27] designed a MEMS switch with four folded springs. The isolation was 28 dB at 10 GHz, and the insertion loss was 0.4 dB at 10G Hz. High isolation of 80 dB at 20 GHz, low insertion loss of 0.4 dB, and low-driving voltage of 2.45 V were achieved in the capacitive MEMS switch designed using serpentine flexure bridge with holes and a dielectric layer made of HfO_2_ with a spring constant of 25 [28]. 

Bridges with low elastic constant were designed in [29,30,31,32] to realize the low-actuation voltage and high isolation of MEMS capacitive switches using folded beams in the millimeter-wave band. 

A serpentine flexure structure with a large series inductance in a MEMS switch was designed by Guha et al. [29], leading to a low-driving voltage of 6.25 V and a high isolation of 70 dB at 40 GHz. A novel capacitive MEMS switch with low insertion loss of 0.29 dB@35 GHz was presented by Li et al. [30] in which the utilizing membrane was connected to the anchors by four springs. Both MEMS capacitive switches proposed by Ma et al. [31,32] achieved high isolation and low-actuation voltage using folded beams in the V band, but due to the small air gaps and small thickness of the CPW line, the switches had excessive insertion loss, which was caused by the conductor and microbridge. Detailed results are shown in Table 1. 

#### 3.1.2. MEMS Switches Based on Combinations of Several Switches in Series in Different Frequency Bands

Some MEMS switches were designed based on combinations of several switches in series to achieve high isolation. A RF signal can be isolated several times by this combination; therefore, the isolation of the MEMS switch is greatly improved. Table 2 summarizes the performance parameters of MEMS switches using this type of combination in different frequency bands.

Both capacitive MEMS switches presented in [25,33] achieved high isolation in the centimeter band by using two identical bridges in series. Comparing the switches with the single bridges in Table 1, the isolation of the switch with double bridges was almost doubled. The dual- bridge structure was used by Tang et al. [25] to improve isolation while sacrificing insertion loss. A high-impedance transmission line was designed by Shajahan et al. [33], and the thickness of the CPW was more than two times the skin depth. As a result, the switch achieved a low insertion loss of 0.15 dB at 20 GHz and a high isolation of 60 dB at 12 GHz. Biyikli et al. [34] achieved a high isolation of 40 dB at 25 GHz using a double-arm cantilever and a low insertion loss of 0.2 dB at 20 GHz by optimizing the dimensions of the cantilever in a DC-contact MEMS switch. The SP4T MEMS switch designed by Singh et al. [35] achieved a high isolation of 51 dB at 20 GHz and a low insertion loss of 0.04 dB at 20 GHz using a structure with a shunt capacitive switch and an ohmic contact switch in series. The capacitive MEMS switches designed in [25,33,35] achieved low-actuation voltages by using serpentine flexure structures. 

High isolation was achieved by using combinations of several switches in series in [36,37,38] in the millimeter-wave band. The MEMS switch proposed by Singh [36] achieved a high isolation of better than 30 dB through secondary isolation by using a combination of two bridges in series and a low insertion of 0.1–0.19 dB by covering a metal layer on the dielectric layer in the Q and V bands. The MEMS switch designed by Singh [37] achieved low insertion loss of 0.14 dB at 32 GHz and high isolation of 70 dB at 32 GHz based on a series–shunt configuration. The MEMS switch designed by Rizk et al. [38] achieved high isolation and low insertion loss in the W band using π-match with two bridges in series.

#### 3.1.3. MEMS Switches with a Float Metal Layer on the Dielectric Layer in Different Frequency Bands 

Table 3 summarizes the performance parameters of MEMS switches with a float metal layer on the dielectric layer in different frequency bands. This structure is the equivalent of two capacitors in series in the up-state, leading to the reduction of the capacitance, and thereby, a low insertion loss was achieved in the centimeter Q, and V bands. This design could also solve the problem of reducing the capacitance in the down-state due to the microscopic roughness of the surface of the dielectric layer, leading to high isolation in different bands.

Angira [39,40] designed low-insertion-loss capacitive shunt MEMS switches using a float metal layer covering the top of the SiO_2_ dielectric layer, and the insertion loss was better than 0.11 dB, and the peak isolation was 55 dB at 10 GHz. Bansal et al. [41] achieved a high down-state capacitance and a low up-state capacitance using a torsion beam structure, and the switch achieved a very high capacitive ration of 1175. Angira et al. [42] designed a new type of capacitive shunt MEMS switch with float metal layers for the X and K bands and showed a low insertion loss of 0.01–0.11 dB at 1–25 GHz. A low-actuation voltage was achieved in [39,40,42] using two actuation electrodes and cantilevers with a low spring constant. The MEMS switch designed by Singh [36] achieved a low insertion of 0.1–0.19 dB at 30–75 GHz by covering a metal layer on the dielectric layer and a high isolation of 59 dB at 36 GHz through secondary isolation by using a combination of two bridges in series.

#### 3.1.4. MEMS Switches Based on T-Match and π-Match in Different Frequency Bands

Table 4 summarizes the performance parameters of MEMS switches based on T-match and π-match. Two short high-impedance sections of transmission line were designed before and after the switch by T-match; these sections behaved as series inductors and provided an excellent match at the design frequency. In the case of π-match, a short section of high-impedance line was used between two shunt switches to result in an impedance match [6]. 

Rizk et al. [38] designed high-isolation MEMS shunt switches based on T-match and π-match on silicon substrates in the W band for the first time. The T-match design showed an insertion loss of 0.1–0.5 dB and an isolation of 21–30 dB in the W band, and its actuation voltage was 30 V. The π-match design showed an insertion loss of 0.3–1.3 dB and an isolation of 30–50 dB in the W band, and its actuation voltage was 30 V. Chu et al. designed a MEMS capacitive switch with a high isolation of 35 dB at 35 GHz, low insertion loss of 0.25 dB at 35 GHz based on GaAs monolithic microwave integrated circuit (MMIC) technology, and T-match in the Ka band [43]. The capacitive type MEMS switch designed by Tsaur et al. [44] realized a low insertion loss of 2 dB at 67 GHz and high isolation of 25 dB at 67 GHz based on a PZT/HfO_2_ multilayered dielectric and π-match. A wideband MEMS switch was proposed by Du et al. [45] using standard BiCMOS technology and π-match, and its isolation was 30–50 dB and insertion loss was 1.2–2.7 dB at 180–250 GHz. 

#### 3.1.5. Other Types of MEMS Switches in Different Frequency Bands

In addition to the above MEMS switches, many other types of switches were developed to achieve excellent insertion loss and isolation. Table 5 summarizes other types of MEMS switch performance parameters in different frequency bands.

Lal et al. [46] achieved low insertion loss and high isolation using a novel CPW configuration with discontinuities in a capacitive MEMS switch, in which a new capacitance and inductance were added in series with the equivalent circuit model. The MEMS ohmic switch designed by [47] realized low insertion loss and high isolation using a cantilever beam with multiple contacts and shunt contacts on the cantilever beam connected with the ground lines in the OFF state, which resulted in an improved isolation, and disconnected with the ground lines in the ON state, which resulted in an improved insertion loss. The MEMS metal-contact switch proposed by Yahiaoui et al. [48] achieved high isolation using a see-saw system, which resulted a high isolation gap. The capacitive MEMS switch with parallel-support beam proposed by Shekhar et al. [49] also obtained excellent insertion loss and isolation performances.

A novel metal-contact MEMS switch with a bidirectional, electrothermal MEMS actuator designed by Zhu et al. [50] achieved low insertion loss of 0.25 dB at 35 GHz and high isolation of 32.5 dB at 35 GHz and also realized low pull-down voltage of 0.3–0.5 V due to the high thermal conductivity and high thermal expansion coefficient of the actuator. Deng et al. [51] achieved a low insertion loss of 0.3 dB at 35 GHz using a λg/4 sector open stub in a capacitive MEMS switch. Chan et al. [52] designed a dimple formed along the signal line on top of a bridge to create good contact and achieve good isolation in the V and W bands. Lee et al. [53] achieved high isolation and low insertion loss in the V and W bands using two-directional motions, and the data is listed above.

Both waveguide switches were designed in [54,55]. A single pole, single throw (SPST) waveguide switch designed by Baghchehsaraei et al. [54] exhibited a low insertion loss of 0.6 dB at 60 GHz and high isolation of 32 dB at 60 GHz utilizing a reconfigurable MEMS surface. A novel, monolithic MEMS waveguide switch proposed by Vahabisani et al. [55] achieved an insertion loss of less than 0.2 dB and an isolation of more than 22 dB at 60–75 GHz by embedding the MEMS multilayer cantilever beams made of silicon/chrome/gold in the waveguide channel, and the beam shape was tapered.

Both the signal line with contact bumps and the tip of the cantilever beam were designed as a fork tip, which minimized their overlapping area, in the paper written by Ghodsian et al. [56], and their MEMS switch realized low insertion loss of 0.36 dB at 77 GHz. Excellent performances of insertion loss and isolation were showed in the W band based on the 0.25 um SiGe–C BiCMOS technology proposed by Ulusoy et al. [57]. 

#### 3.1.6. MEMS Switches for Submillimeter Applications.

Table 6 summarizes the performance parameters of MEMS switches for submillimeter applications.

The highly integrated calibration MEMS switch using silicon micromachining proposed by Jung-Kubiak et al. [58] achieved a low insertion loss of 0.2 dB and high isolation of better than 25 dB at 500–750 GHz. An electrostatic MEMS switch designed by Feng et al. [59] showed an insertion loss of 0.7–2.7 dB and a high isolation of 17–25 dB at 500–750 GHz. The CPW transmission lines were suspended on the substrate, and the input part of the signal line was used as a cantilever in which the tip gradually reduced in width in this design. A submillimeter-wave MEMS-reconfigurable phase shifter using MEMS-reconfigurable surfaces designed by Shah et al. [60] exhibited an insertion loss of better than 3 dB at 500–550 GHz and, attributed by the MEMS-switched surfaces and stubs, was only 0.5–1.5 dB. Shah et al. [61] designed a single-pole, single-throw MEMS waveguide switch based on a MEMS-reconfigurable surface and achieved an insertion loss of 2.5–3 dB and an isolation of 19–24 dB at 500–750 GHz. 

#### 3.1.7. Some Materials Used in MEMS Switches in Different Frequency Bands 

Table 7 summarizes some materials used in different bands. Au was generally used as the conductive material of switch due to its low resistivity and low Young’s modulus, leading to low-actuation voltage and low insertion loss in different bands. However, the low elastic modulus of Au tends to cause the reliability problem of stiction of the switch, resulting in a decrease in reliability; therefore, Cu or alloy were also used as the bridge material of the switch to increase the hardness. Si_3_N_4_ or SiO_2_ were generally used as the dielectric layer of the switch material, but in order to further improve the isolation of the switch in high frequency, materials such as Ta_2_O_5_ and HfO_2_ with high dielectric constants, were adopted. High-resistance silicon and GaAs are used as the materials of the substrate to achieve low insertion loss.

### 3.2. Research on Dielectric Charging of MEMS Switches

MEMS capacitive switches exhibit excellent performance in the field of wireless communications; however, reliability issues are still the bottleneck that limits their commercialization [62]. The main failure of RF MEMS switches is “adhesion” caused by dielectric charging [63,64]. The dielectric charging phenomenon has been studied by a number of researchers since it was first observed by Nguyen et al. [65] in 1998. 

The dielectric charging effect arises from charge injection and the dipole orientation, displacement, and trapping that occurs under the strong electric field during the down-state of MEMS capacitive switches [66]. The electric field, generated by pull-down voltage across the dielectric layer, reaches up to 1–3 MV/cm when the switch is in down-state. A leakage current is produced in the dielectric material by different conductive mechanisms [67]. The charge in the leakage current is captured by the trap in the dielectric material when the bridge contacts the dielectric layer, which causes the charge to accumulate. The electric field, generated by the accumulated charge, will affect the electric force on the driving electrode, resulting in the drift of the pull-down voltage and the adhesion problem.

Yuan et al. [68] found that peak voltage, duty factor, and temperature were significant acceleration factors on charging, whereas frequency had little effect on charging. The steady-state leakage current and the steady-state charge density increase at elevated temperatures, whereas the restoring force and spring constant of the membrane decrease at elevated temperatures; therefore, the switch is more prone to stiction due to dielectric charging the when temperature increases [69]. The dielectric charging was strongly affected by temperature and increased exponentially with temperature, especially with high temperature [70]. The increase of charging temperature assists the charge trapping and the increase of discharging temperature leads to an increase in the magnitude of the bulk discharge current [71]. Zhen et al. [72] studied the impact of humidity on dielectric charging in RF MEMS capacitive switches and found that bulk charging dominated in dry air, but surface charging increased linearly with increasing humidity. Zaghloul et al. [73] found that thinner dielectric films had larger induced surface potential, which lead to higher charge trapping; thus, thicker dielectric films would be better for less dielectric charging. Charges were also found to decay faster in SiN dielectric films deposited on metal layers compared with dielectric films deposited on silicon substrates, and high-frequency SiN dielectric films were more resistant to dielectric charging compared with low-frequency ones [73]. The accumulated charge increased with the dielectric film thickness of the low-frequency material but could not be affected by the thickness of the high-frequency material [74]. The high electric field injection leads to defect generation in low-frequency material [75]. Michalas et al. [76] found that dielectric charging occurred when the actuation voltage had not reached the pull-down voltage and called this phenomenon contactless charging. The contactless charging occurs in gaps between the dielectric layer and the membrane due to the contact surface roughness when the membrane is in the down-state. Palit et al. [77] found that the current density of rough contact was several orders of magnitude greater than planar contact based on a study of the effect of the surface roughness on dielectric charging, which affects the reliability of the switch.

Ultra-NanoCrystalline diamond had been used as the dielectric layer of MEMS capacitive switches in [78,79], and dielectric charging had little effect on the diamond switch because the charging and discharging time constants of the diamond were 5–6 orders of magnitude quicker than the conventional material. Thus, the stiction problem due to dielectric charging was solved when the switch was in the down-state. Switches based on aluminum nitride (AlN) dielectric material presented slow dielectric charging and fast dielectric discharging, which could reduce charge accumulation and increase significantly the reliability of the RF MEMS capacitive switches [80]. Increasing touch surface roughness, reducing the actual contact surfaces, and using material with high hardness could weaken the effect of dielectric charging in MEMS capacitive switches [81]. In order to eliminate the contact between the dielectric and the suspended metal, separation posts located at each corner of an MEMS switch and within the actuation pad were proposed in [52], and the separation posts prevented the problem of dielectric charging. Vaha-Heikkila et al. [82] presented a novel dielectric-less MEMS switch to avoid dielectric charging; polysilicon stoppers were designed to prevent ohmic contact between the electrodes in the down-state. In order to reduce charge accumulation, a novel MEMS capacitive switch, which aligned holes in the membrane to a dielectric post, was proposed in [83], and charge across the dielectric layer was reduced; therefore, dielectric charging was reduced. Li et al. [84] found that a double-dielectric-layer structure could reduce the charge accumulation in the dielectric layer compared to the single-dielectric-layer structure. Marcelli et al. [85] found that applying a bipolar driving voltage could reduce dielectric charging and improve reliability. Chu et al. [86] designed a novel MEMS capacitive switch with no dielectric layer on the bottom actuation electrode, which removed a source of dielectric charging, and the bump was also designed to avoid dielectric charging in the switch.

### 3.3. Research on Reliable Contacts of MEMS Switches

Failures in contacts are the main reasons to limit the application of metal-contact MEMS switches, and the performance of this type of switch is primarily determined by the contact area. Contact stiction and contact degradation are the main failures of MEMS switches. Stiction is caused by cold or hot welding and is primarily related to the contact metal structure and the geometry of the contact area. Stiction caused by cold welding depends on the atomic adhesion forces between the contact materials, and stiction caused by hot welding, which is more important for MEMS switches, is related to the electrical current flowing inside the contact, which causes a rise in temperature that causes small asperities to weld to each other [6,87]. Moreover, if the RF power level is increased, the contact failure is exacerbated by the raised temperature in the contact area [88]. In order to reduce contact failure, satisfy higher power handling capability, and improve lifetime at hot switching conditions, much research has been performed. 

The selection of the contact material is a key for achieving reliable contacts. The most widely used contact material in MEMS switches is gold (Au) [89,90,91], because Au has very low resistivity, high conductivity, and high oxidation resistance. Switches [91] based on a nickel cantilever beam and Au–Au contact have a current handling capability of 150 mA and lifetimes exceeding 1 × 10^9^ cycles. A lateral switching relay structure [90] was designed to provide Au–Au contact with as low as 70 mΩ contact resistance and 0.45 current-carrying ability. But contact wear, deformation, and adhesive failure are prone to occurring due to the low hardness and low melting point of Au [92,93]. Thus, the selection of the electrical contact material and packaging are probably the most critical steps. The contact materials should have high hardness for protection against surface degradation, low resistivity for low contact resistance, and high resistance against chemical corrosion. Many researchers have focused on various contact materials with low roughness, low resistivity, high hardness, and high chemical resistance to contamination and corrosion instead of Au–Au contact.

Strategies to improve hardness usually make use of bimetallic contacts and gold alloying with other metals, such as ruthenium (Ru), nickel (Ni), palladium (Pd), or platinum (Pt), or with other noble metals such as silver. Table 8 summarizes some commonly used contact materials. Kwon et al. [88] showed that the failure point of Au–Au was 1.5 × 10^5^, Au–Pt was 1.6 × 10^6^, and Pt–Pt was 1.4 × 10^7^ under hot switching of 100 mA. The measured contact resistance of Au–Au was about 0.1 Ω, Au–Pt was about 0.25 Ω, and Pt–Pt was about 0.4 Ω. Coutu et al. [94] reported that the minimum average contact resistance of microswitches with Au and Au/(6.3%)Pt alloy contacts were measured at 1.17 and 1.87 Ω, respectively. The “hot-switched” life cycles test results were 1.02 × 10^8^ and 2.70 × 10^8^, respectively. The lifetime of switches with Ir–Ir and Au–Ir contacts were 1.3 × 10^7^ and 1.8× 10^6^, respectively. The contact resistance of Ir–Ir and Au–Ir contacts were 0.79 Ω and 0.49 Ω, respectively, under hot switching of 100 mA [88]. Patel et al. [95] presented a switch with an Au–Ru contact, and the switch achieved a contact resistance of 2.4–1.8 Ω at 90–100 V in open laboratory environments (nonpackaged). Czaplewski et al. [27] designed a MEMS switch with RuO_2_–Au contacts. The lifetime of the switch was 1 × 10^11^ cycles, and the resistance was less than 4 Ω. Metal contact switches with binary alloy (Au/Pd, Au/(3.7 at%)Pd) and ternary alloy (Au/Pt/Cu, Au/(5.0 at%)Pt/(0.5 at%)Cu) contacts were designed in [96]. The switches with binary alloy contacts resulted in contact resistance values ranging from 1 Ω to 2 Ω. The reliability testing presented a 3× increase in switching lifetime compared to switches with Au contacts. The ternary alloy showed about a 6× increase in switching lifetime and contact resistance values between 0.2 Ω and 1.8 Ω.

Gold alloying with other metals can also improve their power-handling capability because they can handle large contact temperatures. Kwon et al. [88] showed that a welding problem of the Au–Ir contact occurred at 1.4 W and at 0.5 W for the Au–Au contact. The power-handling capability of the Au–Ir contact was approximately 2.8 times that of the Au–Au contact. Broue et al. [97] reported that the softening of the contact surface occurred at 3 mW for Au–Au contact and at 4.25 mW for Ru–Ru contact, whereas it occurred at 14 mW for Au–Ru contact.

Contamination by materials such as oxygen, carbon, and organic polymers [88,98] can also lead to failure in contacts. Chen et al. [99] found that contact resistance increased with the number of cycles for alloy films due to the accumulation of contaminants on the contact. The noble metals Ru, Rh, and Pt were more sensitive to the growth of a contamination film compared with Au. Thus, the contamination rate could be changed when these metals are alloyed with Au, and high Au content in the alloy could make the metal less vulnerable to contamination failure. Czaplewski et al. [98] observed that switches with Au–Au contacts failed because of adhesion. Switches with Au–Pt and Au–Ir contact did not fail due to adhesion, but instead show lifetime limitations from carbon accumulation on the contacts. Ag–Ag contacts were vulnerable to corrosion and were difficult to operate in a device fabrication process flow. Therefore, it is necessary to realize a hermetic and reliable packaging to minimize contamination from the environment when the switch is working. Switches in [98] were cleaned and sealed using typical metal-sealed hermetic packaging approaches to prevent contamination. Czaplewski et al. [27] proposed that the catalytic behavior of RuO_2_ film delays or prevents the failure of the switches because of accumulation of carbon at the contacts. Kageyama et al. [100] reported an Au–Au/carbon nanotubes (CNTs) contacts RF MEMS switch, which presented better lifetime cycle than the Au-Au contacts switch. 

In the above studies, gold alloying with other metals (such as Au–Pt and Au–Ir contacts) shows reliable characteristics for better lifetime at hot switching conditions and for better power-handling capability. But it is observed that the contact resistance increased compared with the Au–Au contact. Kwon et al. [88] showed that the measured contact resistance of Au/(6.3%)Pt–Au/(6.3%)Pt was 0.72 Ω and Au/(15%)Pt–Au/(15%)Pt was 1.1 Ω. Yang et al. [101] presented that the measured contact resistance of Au/(7.8%)Ni–Au/(7.8%)Ni was 0.62 Ω and Au/(16.7%)Ni–Au/(16.7%)Ni was 0.88 Ω. In order to reduce the contact resistance, some multi-contact MEMS switches were proposed. Patel et al. [95] reported a novel design capable of achieving mN-level Au–Ru contact; two 0.3-m-thick dimples were designed to form the metal-to-metal contact. The design was capable of achieving low resistance. The contact resistance of 2.4–1.8 Ω at 90–100 V was achieved in open laboratory environments (nonpackaged). Liu et al. [102] designed a metal-to-metal contact MEMS switch based on a Pt–Au micro-spring contact. The thickness of the top contact part was decreased alone. The micro-spring contact alleviated mechanical wear on the contacts. The current handling ability was 150 mA per contact, and the lifetime was 1.2 × 10^6^ cycles for hot switching. Patel et al. [103] designed a MEMS metal-contact switch based on mN-level Au–Ru contact. The contact resistance was 1–2 Ω, the power handing capacity was better than 10 W, and the lifetime was 1 × 10^9^ cycles at 2–5 W of RF power. Chow et al. [104] presented a novel Au–Au contact design employing ball-grid array (BGA) dimples, which limited the actual contact area to a few tens of nanometers in diameter, and the power handling was greater than 1 W under the condition of hot switching in excess of 100 million cycles.

### 3.4. Research on Temperature Intability of MEMS Switches

Although, MEMS switches show high linearity, low loss, and low power consumption [6], many MEMS switches are affected by high-temperature sensitivity, especially at high RF power levels [105]. This sensitivity is especially present in multilayer structures and clamped-clamped structures, leading to a change in the air gap and thus, a change in the up-state capacitance and the pull-down voltage [106]. Mulloni et al. [107] found that double-clamped switches were more vulnerable to temperature compared with cantilever switches. The pull-down voltage variations were more than 40% from 25 °C to 70 °C for the clamped-clamped switches. The variations were 11% in the range of 25–100 °C for the cantilever switches. Matmat et al. [108] found that the drift of pull-down voltage increased with temperature, which accelerated switch failure. The drift of pull-down voltage at 40 °C was 2 V greater than that at 20 °C. Zhang et al. [109] found that pull-down voltage had a strong dependence on temperature. The pull-down voltage varied from 50 V to 77 V in the range of 25–50 °C. In order to reduce temperature sensitivity and residual stress sensitivity of MEMS switches, many efforts have been made.

Nieminen et al. [110] developed a novel MEM capacitor based on an optimized thermal compensation structure to minimize the reaction forces from anchors because of thermal expansion. The geometrical compensation was a square frame around a square-shaped suspended membrane, and the anchoring points were placed in the center of each face of the square. Measurement results verified that the pull-down voltage change was less than 5% when the temperature changed in the range of −30 °C to +70 °C. Mahameed et al. [106] presented a MEMS capacitive switch with a thermal compensation structure using standard thin-film technology. The switch consisted of a suspended square plate with four sides connected to rigid anchors at a confined region; a cutout was defined next to each anchor to prevent direct reaction forces from the rigid anchors to the square plate in the event that the switch was exposed to temperature change. The dielectric layer bottom electrode and the bottom electrode were designed to be symmetrical in order to cause a uniform deformation in the plate. The switch demonstrated 50 mV/°C variation in the pull-down voltage at 25–125 °C.

Reines et al. [111] presented an RF MEMS switched capacitor based on a circularly symmetrical structure with arc-type springs placed between the suspended beam and the anchors, resulting in a pull-down voltage variation of only −50 mV/°C from −5 °C to 95 °C, but −340 mV/°C for standard rectangular devices. The symmetrical structure also reduced the sensitivity to stress gradients. Yang et al. [112] designed compact single-pole, multiple-throw MEMS switches based on a symmetrical, circular switch topology. The circular structure was divided into several identical radial switches to achieve a symmetrical structure, which was insensitive to temperature and stress gradient effects. The switches showed a variation <9 V in their pull-down and release voltages from 25 °C to 95 °C. 

Mahameed et al. [20] designed a novel vertical MEMS capacitive switch, which was not sensitive to thermal stresses based on a lateral thermal buckle-beam actuator design. A suspended plate was connected to two rigid anchors by N-beams. The thermal contraction/expansion of the plate was translated to an in-plane displacement. As a result, there was not displacement in the vertical direction for the suspended plate, and the pull-down voltage maintained a constant versus temperature. The variation of the pull-down voltage was 50 mV/°C from 25 °C to 125 °C. Sun et al. [113] developed a temperature-stable metal contact RF MEMS switch using a thermal buckle-beam structure to reduce the temperature sensitivity. The structure could also weaken the sensitivity to stress gradients on the pull-down voltage. The variation of the pull-down voltage was −120 mV/°C from −20 °C to 100 °C. 

Goldsmith et al. [114] reported that the rate of change in actuation voltage using molybdenum as the mechanical material of MEMS capacitive switches was reduced from 0.1 V/°C to 0.3 V/°C for aluminum to less than 0.03 V/°C. The switches could operate at least 20 billion cycles without failure and work up to at least 150 °C without any buckling. Palego et al. [115] demonstrated that a switch with the molybdenum membrane showed a significantly reduced sensitivity to the change of ambient temperature resulting an actuation voltage that varied by less than 0.035 V/°C, but varied by 0.3 V/°C for the aluminum membrane. Both aluminum and molybdenum switches showed a power-handling capacity of 600 mW, and the lifetime for the switch with molybdenum membrane exceeded 2 × 10^9^ cycles.

Mahameed et al. [116] reported novel MEMS tunable capacitors with low-temperature sensitivity. In the design, a cantilever beam was connected to rigid anchors using two small tethers. These tethers could reduce the initial deflection of the cantilever tip because of residual stress gradients. The two tethers were tilted by an angle from the y-axis to reduce the device temperature sensitivity. The designs presented measured pull­down voltage variation of <50 mV/°C at 20–120 °C.

Patel et al. [103] designed a MEMS metal-contact switch with stress and temperature stability for high-power applications. The switch was designed based on an inverted crab topology with four curved springs and a thick plate. This topology exhibited less sensitivity to stress gradients than fixed-free designs and less sensitivity to temperature and biaxial stress than fixed-fixed designs. The measured pull-down voltage was 71–77 V when the temperatures varied from 25 °C to 125 °C; the release voltage was 71–75 V from 25 °C to 105 °C.

Nadaud et al. [117] designed a thin-film packaged MEMS switched capacitor on silicon substrate. The MEMS capacitive structure was integrated into a protective dielectric shell of silicon nitride, and the actuation electrode was standing above the deflectable membrane. The membrane metal was Ti–Au, and the material of electrode was Al. The performance of the proposed switch was not sensitive to temperature changes. The variation of the pull-down voltage and release voltage was 77 mV/°C from 20 °C to 85 °C.

Demirel et al. [118] designed a novel temperature-tolerant MEMS switch for Ka-band applications. The bridge of the switch was designed with four anchors, which were placed on a wafer and on the ground, and also with sharp corners on the mechanical legs to obtain the desired RF and mechanical performance. The edges were chamfered to reduce bucking. The mechanical legs were extended and folded to get low-actuation voltage and get enough thermal expansion. The structure of the proposed switch could reduce the thermal expansion based out of plane deflection because the bridge could move in the lateral direction. The movement of the bridge in the lateral direction could lower the stress level on the bridge and prevent the permanent deformation of the bridge. The measured pull-down voltage was 22 V and 25 V before and after 200 °C thermal treatment. 

### 3.5. Research about Multiband MEMS Switches

Nowadays, the system becomes more complicated with the development of science and technology, and complex systems need multiband operation. They require many small unit cells, such as RF MEMS switches, for multifrequency band functionality [119]. To overcome these problems, some studies have proposed multiband RF MEMS switches, which can be tuned to different frequency bands by tailoring the switch geometry and size.

**Figure 2:**
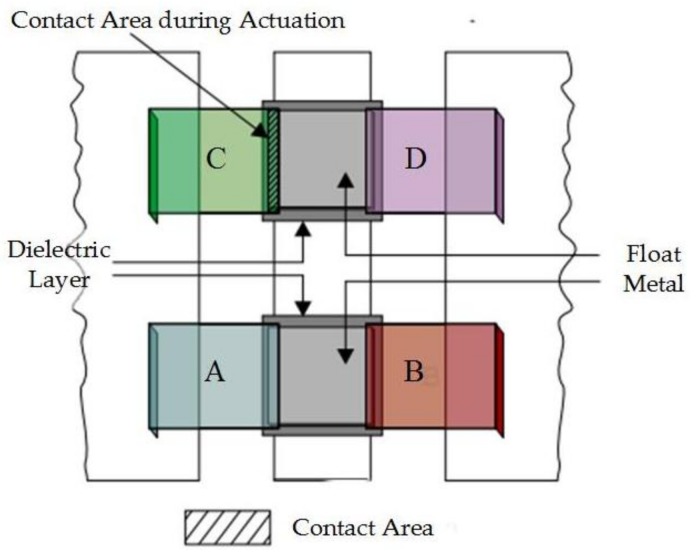
The novel capacitive MEMS switch with high capacitance ratio for multiband. Reprinted by permission from [36].

Singh designed a novel capacitive MEMS switch with a high capacitance ratio for multiband and variable bandwidth [36]. The structure of the proposed switch is shown in Figure 2. “A”, “B”, “C”, and “D” cantilevers mounted on the ground planes on both sides of the switch are designed to be used in various configurations to realize multiband operation. A thin metal layer was deposited over the dielectric layer to solve the reliability problems due to stiction with the dielectric layer. The insertion loss of this switch was 0.1–0.19 dB at 30–60 GHz. When all four of these cantilevers were pulled down, the isolation was better than 30 dB at 30–76 GHz (38 dB at 40 GHz), 30–36 dB at 50–75 GHz, and 26–30 dB at 75–110 GHz. When “AB” or “CD” cantilevers were pulled down, the isolation was better than 30 dB at 30–54 GHz and 50 dB at 40 GHz. When “ABC”, “ABD”, “ACD”, or “BCD” cantilevers were pulled down, the isolation was better than 30 dB at 30–68 GHz and 57 dB at 36 GHz.

**Figure 3:**
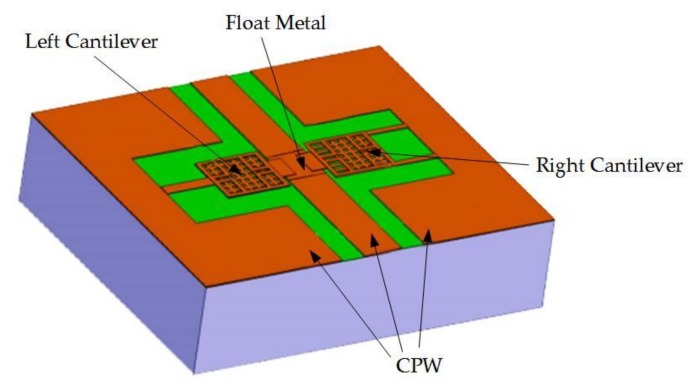
The capacitive MEMS switch with asymmetrical cantilevers. Reprinted by permission from [40].

Angira et al. [40] proposed an asymmetric structure on either side of the transmission line utilizing the float metal concept to implement the switch. The structure is presented in Figure 3. The conventional membrane was replaced by two asymmetrical cantilevers. The novel structure was used to inductively tune the isolation, which enables the switch to be used in the C, X, and Ku bands. When right, left, or both cantilevers were in the down-state, the isolation peaks were 44.22 dB at 8.8 GHz, 42.63 dB at 7.2 GHz, and 47.75 dB at 16.5 GHz, respectively. The insertion loss was better than 0.10 dB, and the return loss was below 23.93 dB up to 25 GHz. 

Angira M et al. designed a new type of capacitive shunt MEMS switch for the X and K bands [42]. The switch structure was implemented with two dissimilar shapes of cantilevers attached to a bridge anchored in between the ground planes of the CPW on either side. Therefore, the inductance value was different on either side of the bridge, which enables the switch to be used in the X and K bands. The insertion loss was 0.01–0.11 dB at 1–25 GHz. When right, left, or both cantilevers were in the down-state, the isolation peaks were 34.33 dB at 11 GHz, 34.71 dB at 10.4 GHz, and 40.7 dB at 21.4 GHz, respectively. 

Puyal et al. [120] designed a frequency scalable MEMS capacitive shunt switch at the millimeter-wave frequency, The switch had different resonant frequencies by changing the size of the switch. When the size of the bridge was 450 μm × 450 μm with the 52–65 GHz operating frequency range of the switch, the insertion loss was 0.6 dB at 60 GHz and the isolation was 3 2 dB at 60 GHz. When the size of the bridge was 400 μm × 350 μm with the 68–88 GHz operating frequency range of the switch, the insertion loss was 0.3 dB at 78 GHz and the isolation was 25 dB at 78 GHz. When the size of the bridge was 350 μm × 340 μm with the 86–102 GHz operating frequency range of the switch, the insertion loss was 0.3 dB at 94.5 GHz and the isolation was 27 dB at 94.5 GHz.

Persano et al. [121] properly modified the design and the fabrication process of MEMS switches, which resulted in devices differing only in the suspended/fixed bridge on the actuator, the presence/absence of the floating metal on the actuator, and the geometric parameters of the bridge, in order to tune the resonance frequency. The resonance frequency measured for the suspended bridge on the actuator presented an increase of around 18 GHz compared with the fixed bridge on the actuator. The tuning of the resonance frequency varied from 23 GHz to 30 GHz with different geometric parameters of the bridge.

## 4. Conclusions

The following conclusions are drawn through the analysis of the above research:

Conclusion 1: Insertion loss and isolation are two critical performance parameters, which are determined by the structures and materials of MEMS switches. MEMS switches have excellent performance and are widely used in low frequency. However, the parasitic effect of the switch is significantly enhanced as the frequency increases, resulting in a drastic deterioration of the isolation performance. At the same time, the skin effect of the conductor is also significantly increased in the high frequency, so the insertion loss performance will be dramatically worsened. MEMS switches with serpentine flexure structures can achieve high isolation and low actuation in the centimeter, Q, and V bands, as well as switches using combinations of several switches in series. However, the latter present higher isolation for high frequency applications. Serpentine flexure structures could reduce the spring constant and increase series inductance of the bridge, therefore high isolation and low actuation voltage were achieved. The RF signal can be isolated several times in condition of combinations of several switches in series, therefore, the isolation of the MEMS switch is greatly improved. MEMS switches with a float metal layer on the dielectric layer demonstrate very low insertion loss and excellent isolation in the centimeter, Q, and V bands. This structure is the equivalent of two capacitors in series in the up-state, leading to the reduction of the capacitance and low insertion loss. Using dielectric layer materials with high dielectric constants, such as Si_3_N_4_ or SiO_2_, and conductor materials with low resistance, such as Au, are also key for the high performance of MEMS switches. T-match and π-match both provide an excellent match at the design frequency. Although, there are fewer studies in submillimeter band, MEMS waveguide switches based on reconfigurable MEMS surface design and BiCMOS technology present good performance in submillimeter applications.

Conclusion 2: The accumulation of charge in the dielectric layer due to a strong electric field will cause the drift of the pull-down voltage, and this even causes “self-locking” of the switch, which seriously affects reliability. The peak voltage, temperature, humidity, thickness of the dielectric film, dielectric material, and the contact surface roughness are significant acceleration factors on charging effects. Dielectric charging increases with increasing temperature, humidity, peak voltage, and thickness of low-frequency dielectric film. However, the charging decreases with the increasing thickness of the dielectric film. Dielectric charging could be reduced by selecting Ultra-NanoCrystalline diamond and aluminum nitride dielectric materials instead of conventional materials. Applying a bipolar driving voltage, separation posts, polysilicon stoppers, and a double-dielectric-layer structure could also weaken charging effects.

Conclusion 3: Contact stiction and contact degradation are the main failures of metal-contact switches related to the contact metal structure and the geometry of the contact area. Temperature rise is the direct cause of stiction. If the RF power level is increased, the contact failure is exacerbated by the raised temperature in the contact area. Au is the most widely used contact material in MEMS switches due to its very low resistivity, high conductivity, and high oxidation resistance, but contact wear, deformation, and adhesive failure are prone to occurring due to the low hardness and low melting point of Au. Strategies to improve hardness usually make use of bimetallic contacts and gold alloying with other metals, such as Ru or Pt, or with other noble metals. Bimetallic contacts and gold alloying show better switch lifetimes at hot switching conditions and excellent power-handling capability. However, noble metals, such as Ru and Pt, are more sensitive to the growth of a contamination film compared with Au; this contamination film makes the switch vulnerable to contamination failure. High Au content in the alloy, hermetic and reliable packaging, and contact material with CNTs could make the metal less sensitive to contamination failure. At the same time, it is observed that the contact resistance of gold alloying increased compared with the Au–Au contact. Multi-contact MEMS switches are proposed to achieve mN-level contact, leading to low contact resistances.

Conclusion 4: Pull-down voltage has a strong dependence on temperature, especially at high RF power levels. The drift of pull-down voltage increased with temperature, and it was especially present in multilayer structures and clamped-clamped structures, which accelerated switch failure. Thermal compensation structures, circularly symmetrical structures, thermal buckle-beam actuators, and inverted crab topology structures have been proven to significantly reduce temperature sensitivity and residual stress sensitivity, but they are complex. At the same time, switches based on molybdenum membrane and thin-film packaging also exhibit less sensitivity to temperature. 

Conclusion 5: Multifrequency band functionality MEMS switches have been achieved by tailoring switch geometry and size. Switches have different inductance and capacitance values due to reconfigurable switch structure and changing membrane bridge size, resulting in different resonant frequencies. Such switches present good performance at these resonant frequencies.

The following methods of improving the performance of MEMS switches in high frequency are proposed according to the above conclusions:

- Utilize combinations of several switches in series and π-match to improve isolation;
- Cover a float metal layer on the dielectric layer, select conductor materials with low resistance, and use matching technology to achieve low insertion loss;
- Design MEMS switches based on serpentine flexure structures and use dielectric layer materials with high dielectric constant to get low pull-down voltage;
- Design MEMS waveguide switches based on reconfigurable MEMS surface and BiCMOS technology to achieve excellent switch performance for submillimeter applications;
- Select Ultra-NanoCrystalline diamond or aluminum nitride dielectric materials and apply a bipolar driving voltage, stoppers and a double-dielectric-layer structure to weaken charging effects;
- Adopt gold alloying with CNTs, hermetic and reliable packaging, and mN-level contact to minimize contact failure; and
- Employ thermal compensation structures, circularly symmetric structures, thermal buckle-beam actuators, molybdenum membrane, and thin-film packaging to reduce temperature sensitivity.

## 5. Summary

In this paper, we introduce the research status of MEMS switches in different bands and of some reliability issues, such as dielectric charging, contact failure, and temperature instability. The performance parameters in different bands and reliability are analyzed to provide research ideas for implementing high-performance MEMS switches in high-frequency fields. Some methods of improving MEMS switch performance at high frequencies are listed. Due to their high isolation, low insertion loss, high linearity, and low power consumption, MEMS switches have great application prospects. However, there are still many technical problems that need to be resolved in order to achieve low insertion loss, high isolation, and high reliability of MEMS switches in high frequencies.

## Figures and Tables

**Table 1:** Comparison of MEMS switches with serpentine flexure structures in different bands.

Band	Insertion Loss	Isolation	Actuation Voltage	Reference
centimeter	0.35 dB@13 GHz	37 dB@10 GHz	3.6 V	[25]
	0.2 dB@15 GHz	23.57 dB@15 GHz	10 V	[26]
	0.4 dB@10GHz	28 dB@10 GHz	-	[27]
	0.4 dB@20 GHz	80 dB@20 GHz	2.45 V	[28]
Q	0.82 dB@40 GHz	70 dB@40 GHz	6.25 V	[29]
	0.29 dB@35GHz	20.5 dB@35 GHz	18.3 V	[30]
V	9.2 dB@55 GHz	23 dB@55 GHz	2.9 V	[31]
	7.25 dB@60 GHz	27.29 dB@60 GHz	3.53 V	[32]

**Table 2:** Comparison of MEMS switches using combinations of several switches in series in different bands.

Band	Insertion Loss	Isolation	Actuation Voltage	Reference
centimeter	0.6 dB@13 GHz	50 dB@9 GHz	3.6 V	[25]
	0.15 dB@12 GHz	60 dB@12 GHz	7.5 V	[33]
	0.2 dB@25 GHz	40 dB@25 GHz	10 V	[34]
	0.04 dB@20 GHz	51 dB@20 GHz	7.19 V	[35]
Q	0.1–0.19 dB	57 dB@36 GHz	-	[36]
	0.14 dB@32 GHz	70 dB@32 GHz	22.5 V	[37]
V	0.1–0.19 dB	30–36 dB	-	[36]
W	0.3–1.3 dB	30–50 dB	30 V	[38]

**Table 3:** Comparison of MEMS switches with a float metal layer on the dielectric layer in different bands.

Band	Insertion Loss	Isolation	Actuation Voltage	Reference
centimeter	0.11 dB@25 GHz	55 dB@10 GHz	11.75 V	[39]
	0.1 dB@25 GHz	47.75 dB@16.5 GHz	6.75 V	[40]
	0.1 dB@20 GHz	43 dB@9.5 GHz	20 V	[41]
	0.11 dB@25 GHz	40.7 dB@21.4 GHz	12.5 V	[42]
Q	0.1–0.19 dB	57 dB@36 GHz	-	[36]
V	0.1–0.19 dB	30–36 dB	-	[36]

**Table 4:** Comparison of MEMS switches based on T-match and π-match in different bands.

Band	Insertion Loss	Isolation	Actuation Voltage	Reference
Q	0.25 dB@35 GHz	35 dB@35 GHz	-	[43]
W	0.3–1.3 dB	30–50 dB	30 V	[38]
	2 dB@67 GHz	25 dB@67 GHz	22–23 V	[44]
180–250 GHz	1.2–2.7 dB	>30 dB	50 V	[45]

**Table 5:** Other types of MEMS switch performance parameters in the centimeter band.

Number	Insertion Loss	Isolation	Actuation Voltage	Reference
centimeter	0.25 dB@20 GHz	58 dB@21 GHz	-	[46]
	0.1 dB@20 GHz	22.3 dB@20 GHz	-	[47]
	0.5 dB@10 GHz	28 dB@10 GHz	15	[48]
	0.25 dB@20 GHz	40 dB@30 GHz	6.2 V	[49]
Q	0.25 dB@35 GHz	32.5 dB@35 GHz	0.3–0.5 V	[50]
	0.3 dB@35 GHz	20 dB@35 GHz	15.8 V	[51]
V	<1 dB	>22 dB	15.2 V	[52]
	1.12 dB@50 GHz	42.2 dB@50 GHz	15 V	[53]
	0.4 dB@60 GHz	35 dB@60 GHz	40–44 V	[54]
	<0.2 dB	>22 dB	-	[55]
W	<6 dB	20–22 dB(Si) 15–30 dB(GaAs)	15.2 V	[52]
	0.36 dB@77 GHz	17.3 dB@77 GHz	39 V	[56]
	2.23 dB@80 GHz	41.4 dB@80 GHz	15 V	[53]
	<1 dB	10–50 dB	40 V	[57]

**Table 6:** The performance parameters of MEMS switches for submillimeter applications.

Number	Band	Insertion Loss	Isolation	Reference
2	500–750 GHZ	0.2 dB	>25 dB	[58]
3	500–750 GHZ	0.7–2.7 dB	17–25 dB	[59]
5	500–550 GHZ	0.5–1.5 dB	-	[60]
6	500–750 GHZ	2.5–3 dB	19–20 dB	[61]

**Table 7:** Some materials used in different bands.

Band	Insertion Loss	Isolation	Dielectric Layer	Substrate	Bridge	Transmission	Reference
centimeter	0.35 dB@13 GHz	28 dB@13 GHz	Si_3_N_4_	Si	Au	Au	[25]
	0.2 dB@15 GHz	23.57 dB@15 GHz	SiO_2_	SIOG	Au	Au	[26]
	0.4 dB@20 GHz	80 dB@20 GHz	HfO_2_	Si	Au	Au	[28]
Q	0.82 dB@40 GHz	70 dB@40 GHz	Si_3_N_4_	Si	Au	Au	[29]
	<0.35 dB	>38 dB	HfO_2_	SiO_2_	Au	Au	[30]
	0.3 dB@35 GHz	20 dB@35 GHz	Si_3_N_4_	Si	Au	Au	[51]
	0.29 dB@35 GHz	20.5 dB@35 GHz	Si_3_N_4_	Si	Au	Au	[31]
	0.25 dB@35 GHz	35 dB@35 GHz	Si_3_N_4_	GaAs	Au	Au	[43]
V	<1 dB	>22 dB	Si_3_N_4_	GaAs	Au	Au	[52]
	<1 dB	>22 dB	Si_3_N_4_	Si	Au	Au	[52]
W	<6 dB	15–30 dB	Si_3_N_4_	GaAs	Au	Au	[52]
	<1 dB	20–22 dB	Si_3_N_4_	Si	Au	Au	[52]
	2 dB@67 GHz	25 dB@67 GHz	PZT/HfO_2_	Si	Au	Au	[44]

**Table 8:** Comparison of some commonly used contact materials.

Numbers	Contact Materials	Contact Resistance (Ω)	Lifetime (cycles)	Reference
1	RuO_2_–Au	4	1 × 10^11^	[27]
2	Au–Au	0.1	1.5 × 10^5^	[88]
3	Pt–Pt	0.4	1.4 × 10^7^	[88]
4	Au–Pt	0.25	1.6 × 10^6^	[88]
5	Au/(6.3%)Pt–Au/(6.3%)Pt	0.72	6 × 10^5^	[88]
6	Au/(15%)Pt–Au/(15%)Pt	1.1	-	[88]
7	Ir–Ir	0.79	1.3 × 10^7^	[88]
8	Au–Ir	0.49	1.8 × 10^6^	[88]
9	Au–Au	1.17	1.02 × 10^8^	[94]
10	Au/(6.3%)Pt–Au(6.3%)Pt	1.87	2.70 × 10^8^	[94]
11	Au/(7.8%)Ni–Au/(7.8%)Ni	0.62	-	[101]
12	Au/(16.7%)Ni–Au/(16.7%)Ni	0.88	-	[101]
